# Development of ASIC1a ligand-gated ion channel drug screening assays across multiple automated patch clamp platforms

**DOI:** 10.3389/fnmol.2022.982689

**Published:** 2022-10-19

**Authors:** John Ridley, Sam Manyweathers, Raymond Tang, Tom Goetze, Nadine Becker, Ilka Rinke-Weiß, Robert Kirby, Alison Obergrussberger, Marc Rogers

**Affiliations:** ^1^Metrion Biosciences Ltd., Cambridge, United Kingdom; ^2^Nanion Technologies GmbH, Munich, Germany

**Keywords:** automated patch clamp, ligand-gated ionotropic receptor, acid-sensitive ion channel, drug discovery, screening assay

## Abstract

Human acid-sensing ion channels (ASIC) are ligand-gated ionotropic receptors expressed widely in peripheral tissues as well as sensory and central neurons and implicated in detection of inflammation, tissue injury, and hypoxia-induced acidosis. This makes ASIC channels promising targets for drug discovery in oncology, pain and ischemia, and several modulators have progressed into clinical trials. We describe the use of hASIC1a as a case study for the development and validation of low, medium and high throughput automated patch clamp (APC) assays suitable for the screening and mechanistic profiling of new ligands for this important class of ligand-gated ion channel. Initial efforts to expand on previous manual patch work describing an endogenous hASIC1a response in HEK cells were thwarted by low current expression and unusual pharmacology, so subsequent work utilized stable hASIC1a CHO cell lines. Ligand-gated application protocols and screening assays on the Patchliner, QPatch 48, and SyncroPatch 384 were optimized and validated based on pH activation and nM-μM potency of reference antagonists (e.g., Amiloride, Benzamil, Memantine, Mambalgin-3, A-317567, PcTx1). By optimizing single and stacked pipette tip applications available on each APC platform, stable pH-evoked currents during multiple ligand applications enabled cumulative EC_50_ and IC_50_ determinations with minimized receptor desensitization. Finally, we successfully demonstrated for the first time on an APC platform the ability to use current clamp to implement the historical technique of input resistance tracking to measure ligand-gated changes in membrane conductance on the Patchliner platform.

## Introduction

Acid-sensing ion channels (ASICs) are proton-gated cation ion channels which are highly sensitive to extracellular acidosis and variably permeable to Na^+^, Ca^2+^ and other cations (Lingueglia and Lazdunski, 2013). To date six different ASIC subunits and splice variants have been described (ASIC1a, 1b, 2a, 2b, 3, and 4) encoded by four genes (Wemmie et al., 2013). ASIC subunits assemble to form homomeric or heteromeric trimers which exhibit different sensitivities to pH activation and modulation by toxins and small molecules, and differential expression across the central nervous system (CNS) and peripheral nervous system (reviewed in Gründer and Chen, 2010; Lingueglia and Lazdunski, 2013; Heusser and Pless, 2021).

ASIC1a is expressed in various regions of the brain, including the hippocampus, cerebral cortex, cerebellum, and amygdala (Wemmie et al., 2013). Elevated neural activity can lower extracellular pH that activates ASIC1a channels, leading to influx of Na^+^ and membrane depolarization which underlies their involvement in synaptic learning and memory and fear conditioning (Gründer and Chen, 2010). Homomeric ASIC1a channels are also permeable to Ca^2+^ so excessive activation can contribute to neurotoxicity (Bässler et al., 2001; Xiong et al., 2004; Chassagnon et al., 2017). Consequently, ASIC1a is implicated as a therapeutic target in neurological indications associated with pathophysiological extracellular acidification such as ischemia, stroke, inflammation, multiple sclerosis and seizures.

Peripheral ASIC1 ion channels are involved in nociception as they are expressed in dorsal root ganglia neurons and peripheral nerve terminals where they respond to inflammatory-mediated changes in extracellular pH (Gründer and Chen, 2010). Involvement of ASIC1 channels in pain is evidenced by effects of peptide toxins; the ASIC1 activator MitTx evokes intense, long-lasting pain in patients envenomated by the Texas coral snake (Bohlen et al., 2011), whilst the ASIC1a inhibitors PcTx1 (from tarantula venom) and Mambalgins (from mamba snake venom) reduce thermal and mechanical hyperalgesia (Mazzuca et al., 2007; Diochot et al., 2012). Clinical validation of ASIC-mediated nociception was obtained by PainCeptor, whose inhibitors PPC 5650 and PPC 5692 reduced heat threshold and mechanical sensitivity and pain biomarkers in Phase I clinical trials (Heusser and Pless, 2021 and references therein).

The interest in ASIC1a channels as CNS injury and pain targets creates demand for reliable assays to support drug discovery efforts. Ion channel drug discovery now relies on high throughput electrophysiology assays as automated patch clamp platforms (APC) have come to replace plate-based imaging assays (Obergrussberger et al., 2022), but the development of APC ligand-gated ion channel assays has lagged behind that of voltage-gated ion channels. Ligand-gated ion channels with rapid activation kinetics and desensitization present specific challenges on APC systems due to low channel expression, the need for rapid liquid addition and effective wash-off of ligands, and loss of responsiveness due to short- and long-term desensitization. We therefore used hASIC1a as an exemplar ionotropic receptor target to validate, optimize and compare fast ligand-gated receptor channel assays across multiple APC platforms, and assess their suitability for drug discovery screening.

The trigger for our project was work from GlaxoSmithKline describing endogenous pH-gated ASIC responses in HEK cells (Gunthorpe et al., 2001). RT-PCR analysis and manual patch clamp biophysics and pharmacology indicated that functional human ASIC1a channels were present in untransfected parental HEK cells. We sought to replicate this finding and try to extend it to use the innate HEK cell ASIC1a receptor channel in APC drug screening assays. This work highlights the advantages and disadvantages of exploiting the endogenous expression of ion channels in common cell expression systems (e.g., Rogers et al., 2016; Borg et al., 2020). For example, the use of HEK cell parentals to express mutated hASIC1a and other human and species ortholog ASIC channels (Kuduk et al., 2010; Wolkenberg et al., 2011; Goehring et al., 2014; Cullinan et al., 2019) may be compromised by contamination from background ASIC1a channels.

Historically, activity of ionotropic and metabotropic neurotransmitter receptors was studied in native systems and isolated cells using single microelectrode current clamp tracking of input resistance in response to presynaptic, bath or iontophoretic application of agonists (e.g., Hiruma and Bourque, 1995). Receptor stimulation triggers ion channel activity that produces a change in membrane conductance (e.g., opening of GABA_*A*_ receptor Cl^–^ channels or activation of GABA_*B*_ G-protein coupled K^+^ channels), which can be monitored by applying small current injections to measure changes in input resistance and membrane conductance. Our interest in demonstrating this traditional technique on APC was triggered by a recent study with a low throughput single well electro-optical version of the input resistance tracking method (Menegon et al., 2017). A field electrode system applied current pulses to populations of heterologous cell lines and membrane potential responses were measured with fast voltage-sensing dyes. We reasoned that input resistance tracking with single cell resolution could be achieved at higher throughput using the current clamp features available on most APC platforms. We used the Patchliner for this proof-of-concept study, enabling comparison of automated input resistance current clamp alongside modern voltage clamp methods of ligand-gated ion channel screening.

Although we confirmed an endogenous HEK cell pH-gated response mediated by hASIC1 and transferred the assay to an APC platform, issues with current expression and reference pharmacology indicated it was not suitable for use in drug discovery screening. Subsequent experiments with human ASIC1a channels stably expressed in CHO cells allowed successful validation of reliable ligand-gated receptor ion channel screening assays on low (Patchliner), medium (QPatch 48) and high throughput (SyncroPatch 384) APC devices. ASIC1a current responses were reproducible and repetitively activated in the same cell using single pipette and stacked tip techniques which minimize agonist exposure time through rapid liquid application and effective wash-off. ASIC1a channels exhibited expected pH sensitivity and were inhibited by a selection of non-selective (Benzamil, Amiloride, Memantine) and selective antagonists (PcTx1, Mambalgin-3). Finally, we used the current clamp capability available on most APC platforms to successfully implement the traditional input resistance/conductance-tracking technique to measure ligand-gated ASIC1a current activation on the Patchliner.

## Materials and methods

### Cell lines and cell culture

The HEK parental cell line (ECACC) was grown at 37°C in 5% CO_2_ in MEM with addition of 10% foetal calf serum and 1% MEM non-essential amino acids and 2 mM L-Glutamine (all from Thermo Fisher Scientific). Cells were plated in Corning cell culture flasks and passaged after reaching 60–80% confluency using GIBCO TrypLE (Thermo Fisher Scientific). For QPatch experiments, aliquots of dissociated cell suspension were plated into T-175 flasks (Corning) and grown as above in a 5% CO_2_ incubator at 37°C for 1–2 days, and in some cases switched to a 30°C incubator for 12–24 h, with plating densities adjusted for the expected doubling time under each condition.

The B-SYS (GmbH) CHO cell line constitutively expressing human ASIC1a was grown by continuous passaging according to vendor instructions. Briefly, cells were grown at 37°C in 5% CO_2_ in Ham’s F12 media with addition of 10% foetal calf serum (both from Thermo Fisher Scientific) and 250 mg/ml of Geneticin selection antibiotic (Thermo Fisher Scientific). Cells were plated in Corning cell culture flasks and passaged after reaching 70–80% confluency using StemPro Accutase dissociation reagent (Thermo Fisher Scientific). For QPatch experiments, aliquots of dissociated cell suspension were plated into T-175 flasks (Corning) and grown in a 5% CO_2_ incubator at 37°C for 1–3 days, with plating densities adjusted for the expected doubling time to avoid excessive confluency.

The Charles River CHO cell line CT6012 was cultured at Nanion Technologies GmbH (Munich) according to vendor instructions. Expression was induced by application of 1 mg/ml tetracycline 3–6 h prior to each SyncroPatch 384i experiment. For harvesting, cells were rinsed twice in HBSS (room temperature) and incubated in TrypLE (Thermo Fisher Scientific) for 7 min at 37°C. Following this, cells were rinsed and triturated 4–6× in cooled HBSS. Cells were allowed to rest at 10°C for 20 min in external recording solution in the Cell Hotel. The cells were kept at 10°C in the Cell Hotel of the SyncroPatch 384i and shaken at 200 rpm prior to use in the experiments.

### Automated patch clamp

Table 1 outlines the external and internal solutions used on each APC platform. External solutions were prepared from concentrated stocks kept at 4°C or room temperature, whereas internal solution stocks were aliquoted and stored at −20°C prior to thawing on the day of each experiment. All solutions were filtered and checked for final osmolality and pH before use.

**TABLE 1:** APC internal and external solutions.

Constituent	External (mM)			Internal (mM)		
	PL	QP 48	SP384i	PL	QP 48	SP384i
NaCl	140	140	140	10	10	10
KCl	4	5	4	50	40	10
KF	–	–	–	60	–	110
KGluc	–	–	–	–	90	–
HEPES	10	10	10	10	5	10
MES*	10	10	10	–	–	–
MgCl_2_	1	1.2	1	–	3.2	–
CaCl_2_	2	1	2	–	–	–
Glucose	5	11.1	5	–	–	–
EGTA	–	–	–	20	3.2	10

All external solutions with pH below 7.0 used for activating ASIC1 responses had MES substituted for HEPES.

#### Patchliner methods

CHO cells were cultured according to vendor instructions (B-SYS GmbH) and harvested using optimized protocols before transfer of the cell suspension to the automated cell hotel on the Patchliner where cells are stored at RT (∼20°C) in a small Teflon vessel and repetitively aspirated every 30 s using a 1 ml plastic pipette tip to prevent aggregation. The cell hotel parameters can be adjusted (interval, speed and volume of aspiration) depending on the cell type to maintain viability for 2–3 h. Cell sealing was conducted in the presence of elevated divalent cations and lowered Na^+^, which were washed off after whole-cell access was achieved and replaced with external solution while cells were voltage clamped at a holding potential of −60 mV. For current clamp recordings, current was injected to achieve a notional membrane potential of 0 mV (using HEKA “gentleswitch” protocol) and a series of incrementally positive current injections (100 to 200 pA, 100 msec) were applied to measure input resistance under control and pH activating conditions.

ASIC1a currents were elicited under voltage clamp at a holding potential of −60 mV by rapid application (and wash-off) of external solution (with or without compounds) of varied pH (typically 7.0. 6.8, 6.5, 6.0, and 5.5 to enable full range of pH activation). Cells were washed three times with external solution between applications of activating external solution to establish a stable baseline. Cells were then stimulated with repeated applications of either pH 6.5 (screening mode) or varied pH (EC_50_), and concentrations of reference compounds were applied before and during each pH stimulus (IC_50_ mode). The timing, volume and application speed of these liquid applications were varied to obtain optimum ASIC1a current activation during repeated activation. A 2 min period of rest in pH 7.4 solution (and pre-incubation in test compound as appropriate) between each pH challenge was used to aid recovery from desensitization and allow pre-equilibration of modulatory compounds.

For the input resistance tracking experiments, cells were held at 0 mV under current clamp and a train of incrementing current injections (from 100 to 200 pA in 20 pA increments, 200 msec step duration) were applied to monitor membrane resistance (using Ohm’s Law V = I*R). This pulse protocol was repeated as cells were exposed to solutions of decreasing pH from 7.3 to 6.0, and changes in membrane resistance were converted into conductance and normalized to the maximum conductance change seen in each cell (G/G_*max*_) to enable construction of a pH EC_50_ curve.

#### QPatch 48 methods

CHO hASIC1a cells were cultured according to vendor instructions (B-SYS GmbH) and HEK cells using standard in-house methods. Both cell lines were harvested using optimized protocols and re-suspended in serum-free media (Gibco CHO-S-SFM from Thermo Fisher Scientific for CHO cells, and Ex-Cell 302 from Merck Sigma-Aldrich for HEK cells) to obtain a suspension of cells with smooth membranes and little debris, before transfer to the automated cell hotel consisting of a 60 ml plastic container (Sophion SB2050) and a magnetic stir bar to prevent cell clumping. An aliquot of cells is then aspirated into a 1 ml microcentrifuge tube and spun down (typically 150 g for 2.5 min) and the supernatant discarded before a user-defined volume of external solution is added by the robot and the cells re-suspended to achieve the desired final cell suspension density before application to primed QPlates. Cell capture, sealing and whole-cell access was achieved using optimized QPatch protocols before currents were recorded using a holding potential of −60 mV. ASIC1a currents were elicited by rapid application (and wash-off) of external solution of varied pH (typically 7.0. 6.8, 6.5, 6.0, and 5.5) to enable full measure of activation around the expected EC_50_ of ∼ 6.5. Cells were washed three times with external pH 7.3 solution (5 μl) between applications of activating acidic solution to establish a stable ASIC1a current baseline. Cells were then stimulated with repeated 3 s applications (5 μl) of either pH 6.5 (screening mode) or varied pH (EC_50_ mode); each ligand application cycle was repeated every 2 min. Each concentration of reference compound was applied to each cell before (i.e., during each pH 7.3 application period) and during each pH stimulus (IC_50_ mode). Thus, each concentration of test compound was pre-incubated with cells for at least 2 min before, and then during each acidic solution challenge, before wash-off in a neutral pH 7.3 solution that contained the next concentration of the same compound. Cells were allowed at least 2 min between applications of acidic solution to enable ASIC1a receptors to recover from desensitization.

#### SyncroPatch 384i methods

CHO cells expressing ASIC1a (Charles River) were induced using tetracycline 3–6 h prior to dissociation into a cell suspension. Cells were kept in the cell hotel of the SyncroPatch 384i with a set temperature of 10°C and shaking of the plastic reservoir at 200 rpm to prevent aggregation prior to addition to the NPC-384 chip for each experiment. NPC-384 chips were filled with internal and external solutions (see Table 1) followed by the cell suspension. Suction was used to capture and seal the cells on the NPC-384 chip using optimized protocols.

Two different liquid application protocols were utilized on the SyncroPatch 384i platform to assess optimum methods to study ligand-gated ASIC1a responses. For recordings of stability and pharmacology using a bolus or “puff” application of ligand, 5 μl of the agonist solution was aspirated and applied to the cell; liquid dispense speed can be set between 1 and 100 μl/s, and a setting of 40 μl/s was used in these experiments. Following agonist addition, 50 μl of the total 90 μl volume in each well is then taken up by the tip to remove most of the agonist solution from the cell. Final recovery of the ASIC1a receptor response is achieved by taking up wash solution into each tip from a reservoir and applying it to individual cells on the NPC-384 chip, washing the ligand away. For stability experiments, pH 6.5 was applied to each cell followed by a wash with pH 7.4 solution and a wait of 140 s to remove agonist and reverse any desensitization, before a second application of pH 6.5 was made. This sequence was repeated 6–7 times on each cell. For pharmacology experiments, ASIC1a responses were evoked 3 times by pH 6.5 application followed by pre-incubation in the inhibitor for 140 s, after which the inhibitor was co-applied with a pH 6.5 stimulus. Cumulative concentration responses were obtained for each well using 3–4 incrementing concentrations of inhibitor. Concentration response curves for each well were constructed and mean ± S.E.M values for IC_50_ are given.

In the second ligand-gated protocol, “stacked puff” recordings were made whereby a single [agonist_wash] experiment was sequentially aspirated into each tip prior to flowing the layered liquid array onto each cell across the NPC-384 chip. To assess the pH sensitivity of ASIC1a responses, pipette tips were loaded with 5 μl of pH 7.4 wash solution followed by 5 μl of activating extracellular solution (pH 7.2 to 4.8). For each sweep, the baseline current was recorded for 1 s in pH 7.4 control solution before the application of the 5-μl activating solution, and then the pH 7.4 wash solution was dispensed with a delay of 1 s to allow for recording of channel opening and desensitization in the presence of ligand before ligand wash-off. Application of the wash solution was followed by aspiration of liquid (50 μl of 90 μl total volume) from each well after a delay of 4 s, before a second wash step with pH 7.4 was performed prior to the application of the next activating pH (interval between stimuli 140 s). Each well received an addition of pH 6.5 solution followed by application of a test pH, and this sequence was repeated a second time to enable data from each cell to be normalized to the control pH 6.5 addition. The pH response curve was constructed across multiple wells with replicates of 48. Pharmacology experiments were performed as described above in “puff” application.

### Chemicals and reagents

All chemicals were of analytical grade and sourced from Merck Sigma Aldrich. Amiloride and Benzamil were purchased from Merck (Sigma-Aldrich), Mambalgin-3 from Alomone Labs, and PcTx-1 from Tocris. A-317567, Amantadine and Memantine were kind gifts from Vernalis (UK), a Hitgen company (see Rogers et al., 2021b).

### Data analysis

Concentration-response xC_50_ curves from Patchliner and QPatch 48 experiments were calculated by plotting %inhibition (antagonists) or activation (pH) data against concentration in Prism software (GraphPad), using the variable sigmoidal dose-response function to fit the Hill equation with theoretical minimum block of 0% and maximum block of 100% and a free constant for Hill slope. Inward current amplitudes were compared to the response to a control pH application (typically pH 6.5) prior to antagonist application or excitation by solutions of different pH to enable calculation of %inhibition or %activation for each cell.

Data was analyzed on the SyncroPatch 384 using DataControl 384 software (Nanion Technologies GmbH, Germany). Concentration response curves were fit using a Hill Equation. Statistical comparison of IC_50_ values for Benzamil was performed using a Student’s *t* test in Igor Pro 8 (Wavemetrics). For statistical analysis of SyncroPatch 384 plate and assay quality we used the Z’ value typical for HTS evaluation (Zhang et al., 1999; Iversen et al., 2006). Z-Factor (Z‘) is calculated using the equation:


(1) $$Z^{\prime }=1-\frac{\left(3\times SD_{Max}+3\times SD_{Min}\right)}{AVG_{Max}-AVG_{Min}}$$

where AVG is the mean and SD the Standard Deviation. The indices Max and Min in the formula for Z’ refer to the control measurements with maximum current (i.e., response to negative control) and the minimum current (i.e., response to positive control), respectively. *Z*’ values > 0.5 are considered to correspond to an excellent assay for screening (Zhang et al., 1999; Iversen et al., 2006).

## Results

We initially sought to determine if the reported endogenous ASIC1a currents in HEK cells would be amenable for development of ligand-gated ionotropic receptor screening assays on an APC platform. Further work to develop, validate and optimize ASIC1a screening assays suitable for drug discovery applications on low, medium and high throughput microfluidic chip and 384 well-based APC platforms was carried out using CHO cell lines stably expressing the human ASIC1a channel.

### Characterizing endogenous acid-sensing ion channels in HEK cells

To quickly confirm a previous publication that HEK cells express endogenous ASIC1a receptor channels and assess the suitability of this cell system for drug discovery screening on APC platforms, we utilized the medium throughput QPatch 48 device. The QPatch uses 48 well microfluidic planar borosilicate chips and coated titanium pipette tips to make whole-cell recordings and sequentially apply small liquid volumes to enable rapid activation followed by slower deactivation (wash-off) of ligand-gated ionotropic responses. HEK cells were voltage-clamped at −60 mV and maintained in external solution at pH 7.4 to avoid steady-state desensitization of ASIC1 receptor channels. Cells were stimulated by external solutions with pH values ranging between 7.0 and 5.5 that evoked rapidly activating inward currents, followed by wash-off in pH 7.4 solution.

**FIGURE 1:**
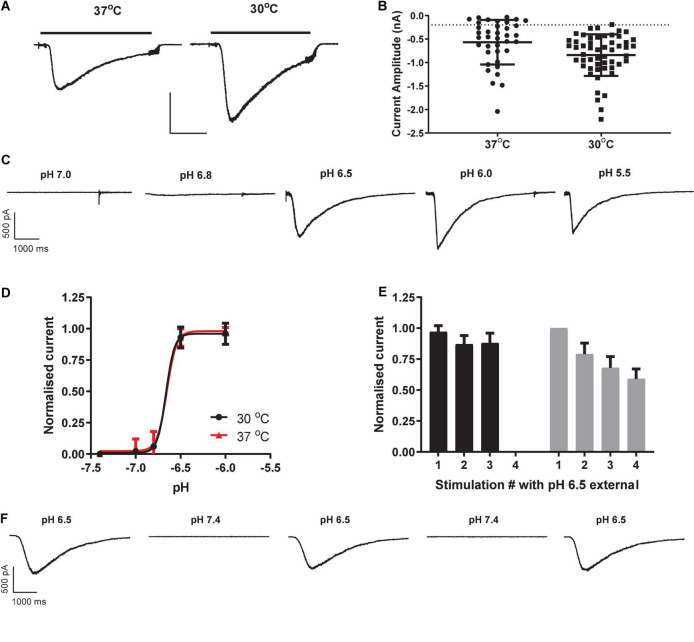
Properties of endogenous proton-activated currents in HEK cells on QPatch 48 APC platform. **(A)** Ligand-gated current responses in cells grown at 37°C or 30°C, showing rapid activation by pH 6.5 solution application (solid bar) followed by slower desensitization and then more rapid wash-off. Scale bar 0.5 nA and 1 s. **(B)** Current amplitude distribution in HEK cells grown at 37°C (*n* = 37) and 30°C (*n* = 53). Mean ± S.D shown by black line and error bars, and minimum current amplitude of –200 pA as dashed line. **(C)** pH-dependency of endogenous proton-activated currents demonstrated by a series of inward current traces in response to rapid application of solutions of increasing acidity. **(D)** Average pH EC_50_ curves from HEK cells grown at 37°C or 30°C (mean ± S.D, *n* = 18 and 26, respectively). **(E)** pH-activated currents in HEK cells grown at 37°C are relatively stable during repeated pH 6.5 stimulation (black bars) but exhibit rundown in cells grown at 30°C to boost current expression (gray bars). Mean ± S.D for *n* = 7 and 11 cells, respectively. **(F)** Stability of inward currents in a HEK cell grown at 37°C during repeated pH 6.5 stimulation, interspersed with application of control pH 7.4 solution.

Consistent with previous manual patch clamp recordings (Gunthorpe et al., 2001), most HEK cells responded to proton challenge on the QPatch 48 with rapidly activating and slowly deactivating inward currents (Figure 1A). As there was a delay in application of the pH 7.4 wash-off solution to each cell, the decay in peak current toward baseline is likely a combination of receptor desensitization followed by ligand removal. Mean inward current amplitude was −567 ± 78 pA (mean ± S.E.M, *n* = 37; Figure 1B), compared to an average amplitude of −334 pA from 47/48 cells in the original manual patch clamp study. The proton sensitivity of endogenous HEK pH-gated currents yielded a pEC_50_ of 6.65 (Figures 1C,D), similar to the published manual patch clamp value of 6.45 (Gunthorpe et al., 2001). The Hill slope of pH activation was very steep (8.2), a common feature of ASIC1 receptor activation attributed to a complex gating mechanism involving proton-binding sites in the extracellular domains which interact with other residues in the acidic pocket and pore (Gründer and Chen, 2010). However, there was considerable variability in the magnitude of pH-evoked responses in HEK cells grown under standard cell culture conditions of 37°C, with a significant fraction (24%) of cells failing to exceed our standard minimum current threshold for APC recordings (dashed line in Figure 1B). APC users frequently assess the success rate (or “patchability”) of an assay by calculating the percentage of cells that pass through each stage of the cell capture and recording process and typical QC filters of seal quality, series resistance. For example, our optimized conditions for endogenous pH-activated responses in HEK cells revealed assay metrics of [100% priming/92% cell-attached/78% seals > 100 MΩ/67% whole-cells/63% completed xC_50_ experiment/48% QC success rate].

In an effort to improve the signal window of endogenous HEK pH-gated responses, cells were incubated for 12 – 48 h at 30°C to induce heat shock and boost protein translocation to the cell membrane. This was moderately successful (Figure 1B), increasing the fraction of expressors to 98% and mean current amplitude to −841 ± 61 pA (mean ± S.E.M, *n* = 53). There was no change in the proton sensitivity of cells grown at 30°C, as the pEC_50_ value of 6.66 at 30°C was identical to that measured in HEK cells grown at 37°C (Figure 1D). Although 30°C treatment improved signal window and initial success rate, there was a corresponding decrease in the stability of pH-evoked inward currents compared to that seen in cells grown at 37°C (Figures 1E,F). We observed significant current rundown during repeated pH challenges in cells grown at 30°C (Figure 1F) leading to poor completion rates of experiments. Best practice in APC ion channel recordings suggests that a rundown rate < 2%/minute is desirable, but various methodological variations and cell biology and biochemical treatments we tested to reduce the rundown rate in cells grown at 30°C to boost current amplitude were unsuccessful (data not shown).

Nevertheless, we sought to confirm that HEK cells express endogenous ASIC1a responses and validate this APC assay using a small toolbox of reference antagonists.

**FIGURE 2:**
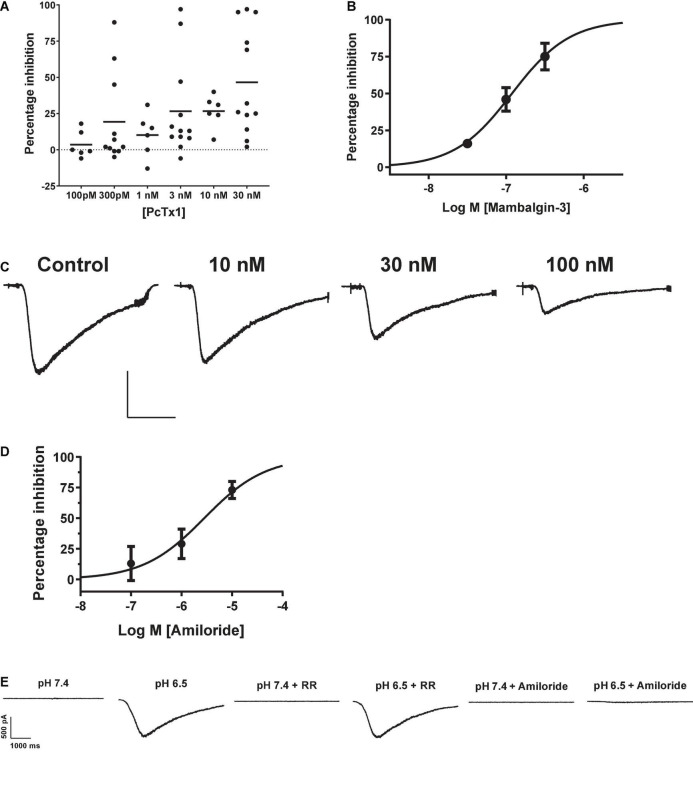
Pharmacology of endogenous proton-activated currents in HEK cells. **(A)** Individual % inhibition values plotted against concentration of PcTx1 applied during QPatch 48 recordings, with mean shown by horizontal black lines. Data was obtained from cumulative IC_50_ assays where each cell was exposed to three concentrations of PcTx1, typically in half log unit increments. **(B)** Potency of Mambalgin-3 to inhibit pH-activated currents plotted as a cumulative concentration-response IC_50_ curve. Symbols are mean ± S.D (*n* = 2 or 3). **(C)** Exemplar current traces from a HEK cell in response to pH 6.5 activation before and after pre-incubation in increasing concentrations of Mambalgin-3. Scale bar is 500 pA and 1 s. **(D)** Inhibition of proton-activated currents in HEK cells by Amiloride. Data was obtained in a similar fashion to C, with each cell stimulated by pH 6.5 solution and then pre-incubated in increasing concentrations of Amiloride to construct a cumulative mini-IC_50_ curve. Symbols are mean ± S.D (*n* = 3). **(E)** Endogenous proton-activated currents are insensitive to Ruthenium Red (RR, 1 μM) but completely inhibited by a non-selective concentration of the ASIC/ENaC/Degenerin antagonist Amiloride (100 μM). The HEK cell was stimulated by pH 6.5 solution and then pre-incubated (at pH 7.4) in RR or Amiloride followed by consecutive stimulation with pH 6.5 solution containing RR or Amiloride.

The tarantula toxin PcTx1 is the most selective antagonist for ASIC1a, with an IC_50_ for the human ortholog of ∼ 3 nM (Cristofori-Armstrong et al., 2019). We observed inconsistent and variable effects of PcTx1 in HEK cells across multiple experimental runs (including in the presence of BSA to avoid protein adherence to glass and plastic surfaces). Many cells showed < 20% inhibition of proton-gated currents by concentrations of PcTx1 from 100 pM to 30 nM (Figure 2A), and mean data fell below 50% inhibition at the highest concentration tested of 30 nM so no IC_50_ value could be reliably extracted from this data. PcTx1 also potentiates ASIC1b and ASIC1a/2a channels under certain conditions (i.e., desensitizing acidic pH) with ∼10 fold lower potency than ASIC1a inhibition (Liu et al., 2018; Cristofori-Armstrong et al., 2019), but we saw no evidence for such modulation in our experiments on endogenous pH-evoked responses in HEK cells.

The green mamba snake toxin Mambalgin-3 is a moderately selective inhibitor of ASIC1-containing receptor channels with an IC_50_ potency of 127 nM for human ASIC1a and 17 – 55 nM for the rat ortholog (Diochot et al., 2012), and its potency against other ASIC homomers and heteromers ranges between 50 and 250 nM (Baron et al., 2013). The endogenous proton-activated currents in HEK cells were effectively inhibited by Mambalgin-3, with ∼75% block at 300 nM and a cumulative IC_50_ value of 119 nM on the QPatch 48 (Figures 2B,C). Mambalgins can also potentiate ASIC1b channels under certain (i.e., acidic) conditions (Cristofori-Armstrong et al., 2021), but we saw no evidence for such modulation in our experiments in HEK cells.

Surprisingly, the benchmark non-selective ASIC1a antagonist Amiloride proved to be a relatively potent but variable inhibitor of pH-evoked responses in HEK cells, frequently exhibiting antagonism at sub-μM concentrations. For example, in some cells 300 nM Amiloride produced ∼25% inhibition of pH-evoked current, whereas in other cells there was no effect until Amiloride concentrations exceeded 10 μM. The mean IC_50_ value obtained for Amiloride in HEK cells was 2.82 μM (Figure 2D), and proton activated currents were completely abolished by 100 μM Amiloride (Figure 2E). Gunthorpe et al. (2001) also reported a relatively high affinity of 2.2 μM for Amiloride inhibition of endogenous pH-gated responses in HEK cells using manual patch clamp. Our and their values are higher than those typically reported for Amiloride inhibition of heterologously expressed hASIC1a, which range from 10 to 30 μM (Leng and Xiong, 2013, and see below). Together with the weak and variable PcTx1 efficacy, these observations suggest that an additional pH-gated ionic conductance may be functionally expressed in HEK cells which could contaminate responses attributed to ASIC1 receptor activation.

TRP channels represent another prominent pH-gated ligand-gated ionotropic receptor family, some of which are expressed endogenously in HEK cells (Wu et al., 2000). Application of 1 μM Ruthenium Red, a non-specific inhibitor of a wide range of TRP channels, failed to significantly reduce the amplitude (12 ± 7% inhibition) of currents activated by pH 6.5 in HEK cells (Figure 2E), suggesting little or no endogenous TRP receptor function under our experimental conditions.

We conclude from these pharmacology experiments using both broad spectrum and more selective ASIC1 antagonists that the endogenous pH-gated response in HEK cells cannot be clearly and unequivocally attributed to ASIC1a (or TRPx) channels, even though ASIC1a is the only member of the ASIC family detected by PCR (Gunthorpe et al., 2001). Along with issues of low current expression and assay stability of the endogenous pH response in HEK cells, all further ASIC1a APC assay development and validation was carried out using CHO cell lines stably expressing sequence-verified hASIC1a, as there is no endogenous pH-gated response in CHO cells and they also lack Amiloride-sensitive EnaC currents (Meltzer et al., 2007; Hoagland et al., 2010).

### Ligand-gated ASIC1a assays on Patchliner automated patch clamp platform

We started development and validation of an APC ligand-gated ASIC1a ion channel assay on the 4 channel Patchliner device as it enables flexible user interaction and cost-effective scale-up from manual patch systems. The Patchliner is a low throughput multi-well APC platform utilizing microfluidic planar borosilicate chips to record whole-cell currents and coated metal pipette tips for solution and compound application. A large range of liquid volumes (10 – 250 μl) can be added to the inflow channels at various speeds (from 1 to 857 μl/sec, typically 4 – 171 μl/sec) to optimize ligand-gated ionotropic receptor activation and wash-off under whole-cell voltage or current clamp. The Patchliner also offers the opportunity to compare and optimize ligand-gated assays using conventional, sequential pipette tip liquid applications or stacked-tip applications.

Initial experiments using sequential single pipette additions of external solution followed by acidic pH solutions elicited rapidly activating ASIC1a currents (−1.0 to −10 nA amplitude, single hole/cell recordings) followed by slower mono-exponential decay back to baseline due, suggestive of receptor desensitization rather than agonist wash-off (data not shown), consistent with previous ligand-gated receptor ion channel experiments on the Patchliner (Nanion, 2013). Such desensitizing ligand-gated ionotropic receptors can make it difficult to distinguish if true peak current (activation) is achieved and resolvable by measurements of maximum amplitude, or if rapid desensitization has begun to erode the peak and affect estimates of agonist EC_50_ and inhibitor IC_50_ values.

**FIGURE 3:**
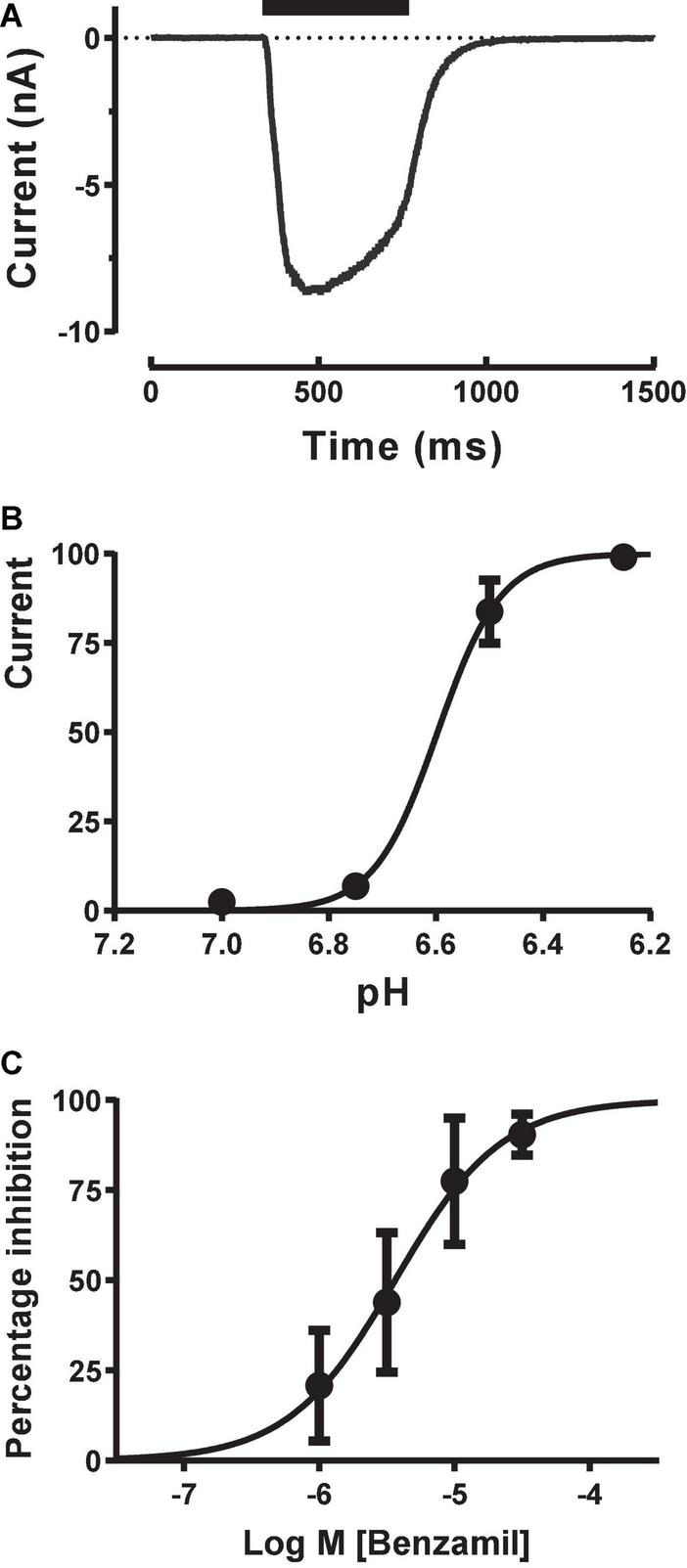
Pharmacology of CHO ASIC1a currents using stacked tip application on Patchliner. **(A)** Rapid activation of inward current during pH 6.5 application, followed by desensitization and then rapid wash-off. **(B)** pH activation reveals an EC_50_ of 6.6 ± 0.01 (mean ± S.D, *n* = 3) with a Hill slope of 7.3. **(C)** ASIC1a current inhibition by Benzamil. Cumulative application of increasing concentrations of Benzamil before and during repeated stimulus with pH 6.5 solution reveal an IC_50_ of 3.65 ± 1.18 μM (mean ± S.D, *n* = 3).

To obtain more reliable estimates of ASIC1a activation and modulation we switched to using stacked tip applications. Each pipette took up a set volume of external solution (wash), agonist (pH solution) and external solution (baseline) and then applied these sequentially in reverse order in a continuous manner to each cell under voltage clamp to rapidly evoke inward currents with minimal desensitization. Starting with the recommended settings from the manufacturer (agonist and wash volumes of 50–200 μl applied at speeds of 12–20 μl/sec), we adjusted the volume and speed of each liquid component application to obtain rapidly activating ASIC1a inward currents which exhibited a clear peak before a slower phase of desensitization occurred, followed by a rapid wash-off back to baseline (Figure 3A). These pH-evoked responses were stable over multiple applications, allowing the construction of cumulative EC_50_ and IC_50_ concentration-response curves. The pH EC_50_ for proton activation was 6.65 with a Hill slope of 7.3 (Figure 3B), and the IC_50_ for Benzamil inhibition was 3.65 μM (Figure 3C), in accordance with literature values for human ASIC1a (Leng et al., 2016).

### Input resistance measurements of ASIC1a activation on the Patchliner

Most marketed APC platforms now offer the option to carry out current clamp recordings, but this capability has not been widely used for drug discovery screening apart from experiments to record action potentials from iPSC cardiomyocytes and neurons (Stoelzle et al., 2011a,b; Haythornthwaite et al., 2012; Goversen et al., 2018; Li et al., 2019, 2021; Becker et al., 2020). We therefore sought to apply a historical current clamp method used to study ligand-gated ionotropic receptor responses to a modern APC platform, utilizing the same CHO ASIC1a cell line employed in our voltage clamp experiments.

**FIGURE 4:**
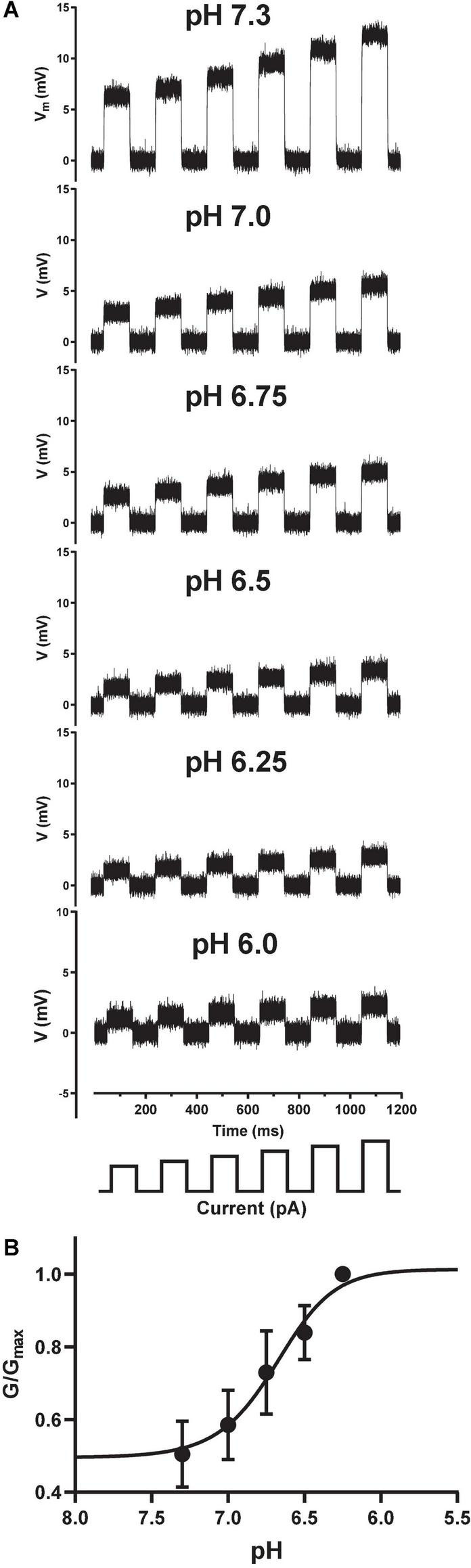
Current clamp input resistance tracking of ASIC1a activation on the Patchliner. **(A)** Under control conditions (pH 7.3), positive current injections of 100 to 200 pA (in 20 pA increments, schematic at bottom) from a holding potential of 0 mV elicit membrane depolarizations. Input resistance in response to the same current injection protocol is gradually decreased after application of increasing pH solutions indicated in each panel. **(B)** Converting membrane resistance to conductance and normalizing the change in conductance for each pH concentration by the maximum conductance change (G/G_*max*_) in each cell allowed plotting of the pH activation curve obtained under current clamp to yield an EC_50_ value of 6.68 ± 0.01 (mean ± S.D, *n* = 3).

Traditional sharp microelectrode and whole-cell manual patch clamp recordings would apply activating ligand by iontophoresis or bath perfusion, and measure changes in passive membrane resistance during agonist exposure by applying a train of small current injections under current clamp. The size of the membrane voltage excursions in response to a set current injection should decrease as channels open and membrane conductance increases and resistance decreases, in accordance with Ohm’s Law (V = I*R). We successfully transferred this ligand-gated current clamp assay paradigm to the Patchliner by holding CHO cells at a membrane potential positive to the net reversal potential for cation flux through the pore, and applied a series of small, incrementing current injections to measure input resistance (Figure 4A). Under control conditions (pH 7.3) this protocol elicited large depolarizing membrane potential responses, which were reduced in amplitude when solutions of increasingly acidic pH were applied to individual cells under current clamp. Using this technique, we could then simply convert membrane resistance to conductance and plot these values against pH to obtain a pH EC_50_ value of 6.68 for ASIC1a receptor channel activation (Figure 4B), identical to the value obtained using voltage clamp (Figure 3B).

### Medium throughput ASIC1a automated patch clamp assay on QPatch 48

Like the Patchliner, the QPatch 48 is a microfluidic planar chip-based APC platform that utilizes metal pipette tips to rapidly add and remove solution from individual wells containing single or multiple cells under whole-cell patch clamp. The QPatch 48 used in this study does not offer the ability to stack solutions in the tips (this has subsequently been introduced to newer QPatch systems), so our challenge in implementing a ligand-gated ASIC1a ion channel assay was the coordination of the scheduling of pipette movements and liquid additions so that agonist solutions are applied quickly and consistently for each cell, and acidic solutions were then washed off effectively to avoid desensitization and current rundown. We therefore limited our ASIC1a experiments to a single “insert” of 16 QPlate wells, and designed the experimental timings to optimize the speed and consistency of liquid additions for each cell or well.

**FIGURE 5:**
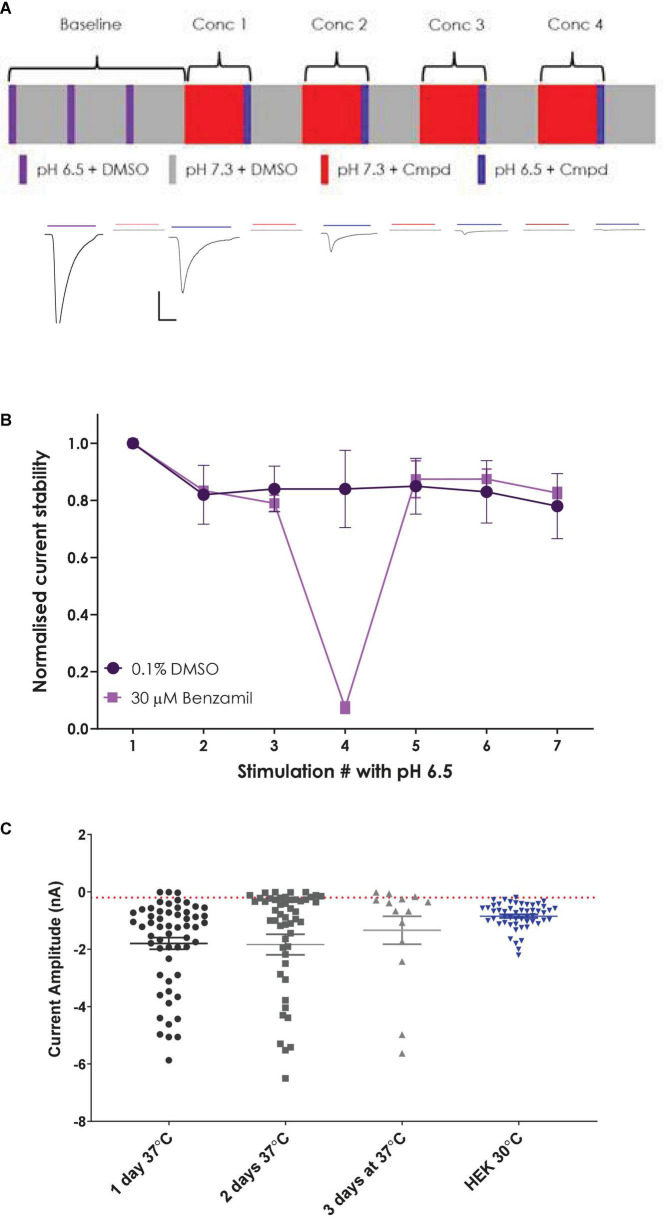
Establishing a stable and robust CHO hASIC1a assay format on QPatch 48. **(A)** Schematic of liquid applications and pre-incubations for a cumulative xC_50_ assay format (upper panel) and exemplar current traces with color-coded application bars (lower panel). Scale bar is 500 pA and 1 s. Minimum and maximum period between applications was 2–3 min. **(B)** Normalized current amplitude during repeated pH 6.5 stimulations in 0.1% DMSO vehicle (circles) or interspersed with a 30 μM Benzamil pre-incubation (squares). Mean ± S.D from *n* = 7 and 2 cells, respectively. **(C)** Comparison of ASIC1a current expression in stable CHO and parental HEK cell lines. CHO cells grown at 37°C for 1–3 days, and HEK cells grown at 30°C. Mean ± S.E.M whole-cell current values shown by whisker graph. Red line is minimum QC current (–200 pA).

Using similar voltage clamp and liquid handling protocols to the initial HEK cell QPatch 48 experiments (except for a small change to pH 7.3 control solution), we established a stable CHO hASIC1a cell response by implementing three pH 6.5 application cycles to obtain a stable current response before applying a four-point cumulative xC_50_ screening paradigm (Figure 5A). Peak current amplitude decreased slightly between the 1st and 2nd pH 6.5 application and then maintained a stable level (and current kinetics) as a steady-state was achieved during subsequent pH activation (Figure 5B). We surmise that steady state current reflects equilibrium between ASIC1a receptor activation, desensitization, and recovery back to the resting closed state after agonist wash-off during each pH challenge cycle, the latter achieved using three washes in pH 7.3 solution and a 2 min recovery period at pH 7.3 between each agonist application.

In contrast to untransfected HEK cells, the stable hASIC1a CHO cell line exhibited robust current expression when cells were grown at 37°C and did not require low temperature treatment to achieve the desired 1–2 nA peak current amplitude or > 50% expressor level that is optimal for APC recordings (Figure 5C). Mean current amplitude was −1.8 ± 0.2 nA (mean ± S.E.M, *n* = 56) after 1 day at 37°C, increasing slightly to −1.84 ± 0.36 nA (*n* = 52) after 2 days in culture and decreasing to −1.34 ± 0.49 nA (*n* = 14) after 3 days at 37°C. Optimal % expressor levels (> −200 pA minimum current) were seen after 1 day *in vitro*.

**FIGURE 6:**
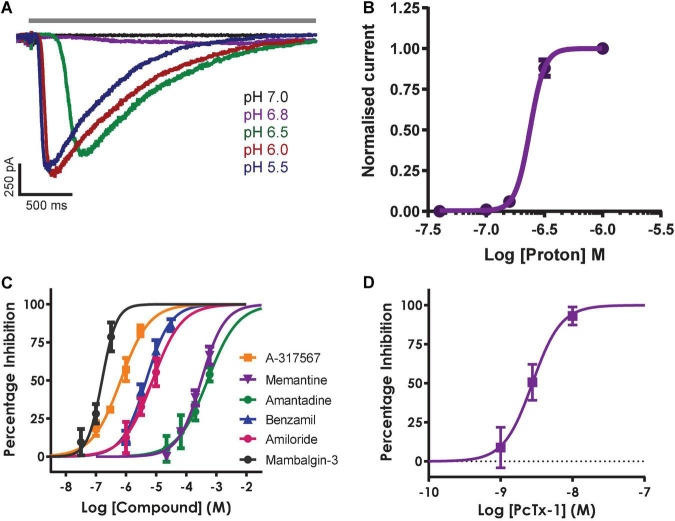
Pharmacology of hASIC1a responses in CHO cell line on QPatch 48. **(A)** Current traces in response to application of solutions (solid gray bar) with indicated acidity. **(B)** pH activation curve indicates an EC_50_ of 6.62 (mean ± S.D, *n* = 5). **(C)** Concentration-response curves for indicated reference antagonists spanning 5 log orders of magnitude. **(D)** Concentration-response data for PcTx1 inhibition indicates an IC_50_ of 2.96 nM. Mean ± S.D, *n* = 3–6 for each compound in **(C,D)**.

Inward currents evoked by application of solutions with pH ranging from 7.0 to 5.5 activated with increasingly rapid kinetics and amplitude followed by a slow phase of desensitization during the 3 s agonist epoch (Figure 6A) and recovery back to baseline after ligand wash-off. Mean data indicate a pH EC_50_ of 6.62 (equivalent to 219 nM H^+^) with a steep Hill slope of 6.9 typical of the co-operative activation of ASIC1 receptor channels (Figure 6B); these values are in good agreement with the literature and results obtained by others on manual and APC platforms (Gunthorpe et al., 2001; Borg et al., 2020; B’Sys, 2022).

The remaining QPatch 48 pharmacology experiments were conducted using pH 6.5 extracellular solution as the agonist to moderately activate ASIC1a currents, as it is close to the calculated EC_50_ value. In addition, this protocol is designed to avoid tachyphylaxis associated with a more acidic pH stimulus, and it allows the development of a ligand-gated ASIC1a channel assay that can distinguish between agonists, partial agonists and antagonists. We used a diverse toolbox of ASIC1 inhibitors to pharmacologically validate the QPatch 48 CHO hASIC1a assay. Reference compounds included the small molecule inhibitors Amiloride, Benzamil, Memantine, Amantadine and A-317567, and the selective peptide toxins Mambalgin-3 and PcTx1 (Dubé et al., 2005; Leng et al., 2016; Nagaeva et al., 2016). Compounds were tested using the cumulative concentration-response assay format shown in Figure 5A, which includes a 2 min pre-incubation period for each concentration to allow test samples to reach and equilibrate with their binding site(s) in the pore or large extracellular binding domain of ASIC1 receptor trimers. Agonist effects can also be detected during this period as membrane current data is recorded after every liquid addition. Figure 6C summarizes the potency of reference compounds against hASIC1a responses on the QPatch 48, covering 5 orders of concentration. Mean inhibition data for each test sample is plotted against Log[compound] and fit with a concentration-response function. The half-maximal inhibitory (IC_50_) values and Hill slopes derived from the fits are listed in Table 2, which are consistent with values obtained from the literature.

**TABLE 2:** ASIC1a pharmacology on QPatch 48.

Compound	IC_50_ potency (μM)	Hill slope	Literature IC_50_ (μM)	References
Amiloride	7.62	0.9	13.5	Leng et al., 2016
Benzamil	4.66	1.1	3.50	Leng et al., 2016
A-317567	0.66	0.9	2.00	Dubé et al., 2005
Amantadine	510.4	0.9	25% at 500 μM	Nagaeva et al., 2016
Memantine	315.3	1.1	31% at 500 μM	Nagaeva et al., 2016
PcTx1	0.003	2.2	0.0032	Cristofori-Armstrong et al., 2019
Mambalgin-3	0.16	1.8	0.127	Diochot et al., 2012

IC_50_ values and Hill slopes derived from the concentration-response fits in Figures 6C,D are compared against values reported from the indicated references.

The non-selective degenerin family antagonists Amiloride and Benzamil inhibit hASIC1a responses on the QPatch 48 with expected potencies between 5 and 10 μM (Table 2), in contrast to the higher potency of Amiloride against the endogenous proton-activated response in HEK cells (∼2 – 3 μM). A-317567 is a small molecule originally developed by Abbott and found to be a more potent antagonist of ASIC3 and ASIC1a channels (Dubé et al., 2005; Kuduk et al., 2010). We found that this compound worked well on the QPatch 48 and therefore it was used as the positive control as it routinely produced potent and complete inhibition, with an IC_50_ of 660 nM; a close analogue of A-317567 developed by Merck and Co., Inc., (Kuduk et al., 2010) was found to inhibit ASIC1a currents with an IC_50_ of 450 nM. Various low molecular weight hydrophobic amines have been found to modulate ASIC receptor channels, including the clinical drugs Amantadine and Memantine (Nagaeva et al., 2016; Shteinikov et al., 2018). We confirmed their low potency inhibition of ASIC1a with IC_50_ values of 510 and 315 μM, respectively (Table 2).

Finally, the QPatch 48 ASIC1a APC assay successfully replicated the published species-specific potency of the hASIC1a-preferring spider venom peptide PcTx1 and the pan-ASIC1 snake toxin Mambalgin-3. The PcTx1 IC_50_ of 2.96 nM we report here (Figure 6D) exactly matches that obtained against hASIC1a in *Xenopus* oocytes by Cristofori-Armstrong et al. (2019), and the Hill slope of 2.2 (Table 2) is consistent with cryo-EM binding and modeling studies that indicate multiple interactions with distinct residues in the acidic pocket and “thumb” regions of the extracellular ligand-binding domain (Cristofori-Armstrong and Rash, 2017). Similarly, the IC_50_ of 160 nM found for Mambalgin-3 inhibition of hASIC1a on the QPatch 48 is very close to the value of 127 nM reported for human ASIC1a in the original publication on this family of snake toxins (Diochot et al., 2012). Importantly, the Hill slopes of the IC_50_ fits for all the reference compounds tested on the QPatch 48 match expected results, with all but PcTx1 exhibiting a non-cooperative binding mode (Table 2) in accordance with previously published data. Overall, this pharmacology data confirms that the QPatch 48 APC ASIC1a assay is capable of accurately identifying antagonists across a wide range of potencies and modalities.

Most APC platforms have the option of using multi-hole plates or chips, where 4–10 apertures are present in each well to allow recordings from multiple cells in parallel. This technique is usually used when target ion channel expression is low (as it sums individual cell current levels), or the assay quality is compromised by issues such as current rundown or other variable parameters. However, multi-hole recordings can also be used to produce a high success rate assay which is very useful when carrying out a HTS with *n* = 1 or *n* = 2 samples, as we did recently for an ASIC1a fragment library screen on the QPatch 48 (Rogers et al., 2021b).

**FIGURE 7:**
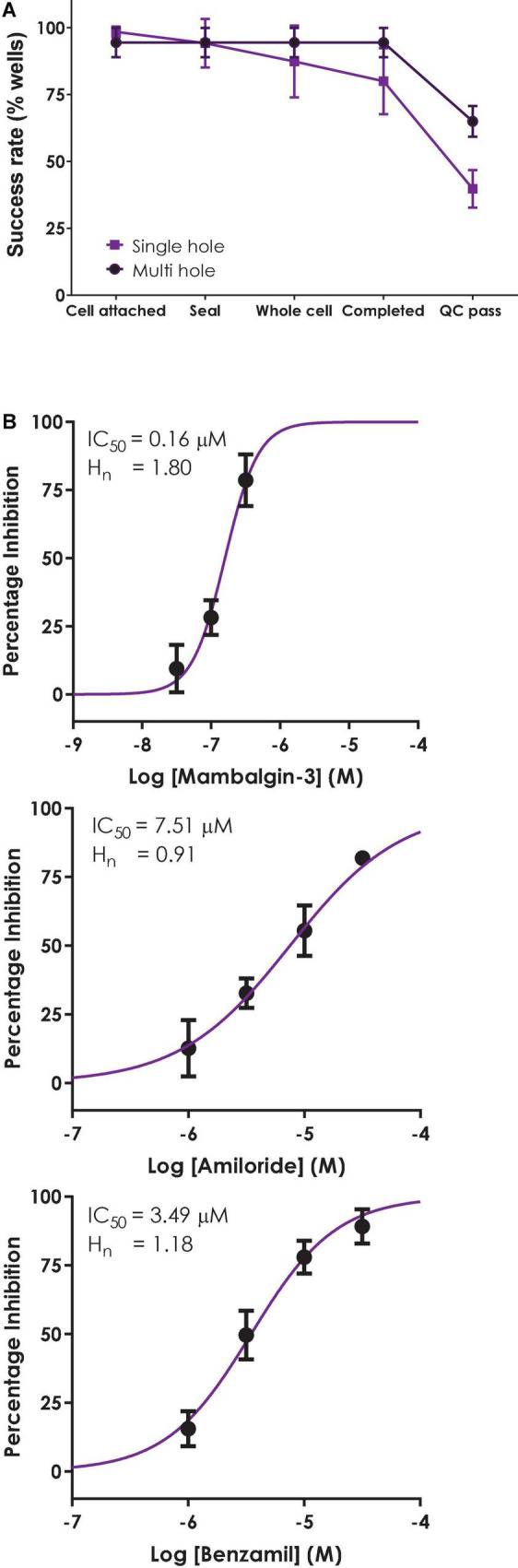
Validation of a multi-hole ASIC1a assay on QPatch 48. **(A)** Comparison of efficiency and success rate of single vs. multi-hole QPlate assay format. Mean (± S.D) data for the percentage of wells that passed various quality control (QC) criteria during and after whole-cell recordings in single hole (*n* = 4 plates, black squares) or multi-hole mode (*n* = 5 plates, filled circles). **(B)** ASIC1a assay pharmacology in multi-hole mode. Mean (± S.D) concentration-response data for inhibition of ASIC1a currents by Mambalgin-3 (*n* = 12), Amiloride (*n* = 8) and Benzamil (*n* = 4) in multi-hole recordings, with indicated IC_50_ and Hill slope values.

Although the single hole/single cell ASIC1a assay on QPatch 48 exceeded our 50% minimum cut-off level in terms of completed recordings (Figure 7A), the final QC pass rate fell below this level due to cells failing our strict QC criteria (−200 pA minimum current, Rseal > 200 MΩ and Rseries < 15 MΩ and stable during the 12–20 min whole-cell recording). In multi-hole mode there was a noticeable increase in the success rate for all parameters to > 90% during the different stages of the experiment, and a significant improvement in the final QC rate to 65%.

Before employing a more efficient multi-hole assay it is important to establish that this variant of the APC whole-cell recording format returns the same biophysics and pharmacology as single cell recordings. To this end we ran some of the reference compounds detailed above in 10 hole “X-plate” mode on the QPatch 48. The potency of the small molecule antagonists Amiloride and Benzamil and the peptide toxin Mambalgin-3 obtained in multi-hole recordings were in very good alignment with values obtained in single hole/single cell mode (Figure 7B and Table 2), confirming the equivalence of each APC recording format.

### High throughput ASIC1a assay on SyncroPatch 384i

The SyncroPatch 384i (SP384i) employs a different design format to the Patchliner and QPatch 48, with plastic tips dispensing liquid straight into individual wells rather than through microfluidic flow channels. While this well-hole layout may not affect drug screening against voltage-gated targets, it may influence ligand-gated recordings in experiments with rapidly activating ionotropic channels prone to desensitization, and in multi-hole recordings receiving liquid applications from a single tip onto multiple cells in the same well, so we also validated the hASIC1a assay on the SP384i.

**FIGURE 8:**
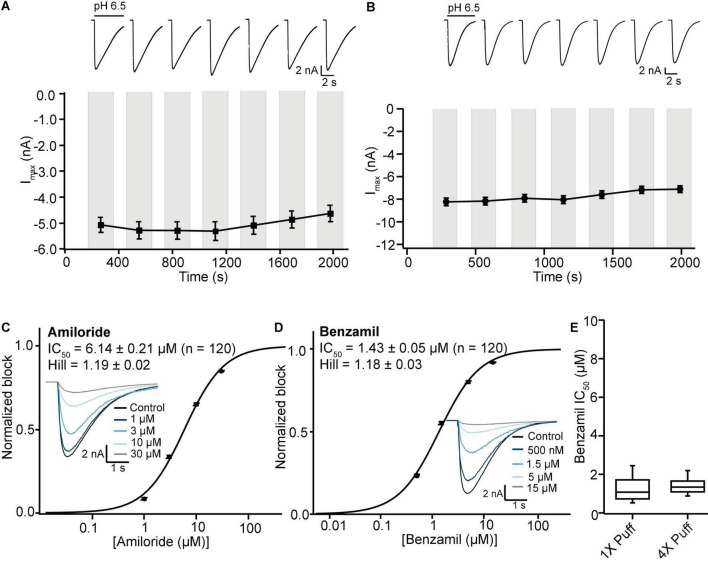
Stable ASIC1a responses in single and multi-hole recordings on SP384i enable pharmacological screening. **(A)** Stable responses in a single hole well to repeated “puff” applications of pH 6.5 solution (top), and average current stability data from *n* = 50 wells (bottom; mean ± S.E.M). **(B)** Stable responses in a multi-hole well (4X) to repeated “puff” applications of pH 6.5 solution (top), and average current stability data from *n* = 60 wells (bottom; mean ± S.E.M). **(C,D)** Inhibition of ASIC1a responses on SP384i by Amiloride and Benzamil. Raw current traces (insets) in response to pH 6.5 “puff” activation after pre-incubation in indicated concentrations of drug enable construction of cumulative IC_50_ curves for Amiloride **(C)** and Benzamil **(D)**, with indicated mean, S.E.M and *n* values and Hill slopes of 1.19 and 1.18, respectively. Multi-hole chips (4X) were used. **(E)** There is a wider spread of IC_50_ values for Benzamil on 1X chips compared with 4X chips using the “puff” ligand application method, although the mean values were not statistically different (*P* > 0.05, unpaired Student’s *t* test).

Initial SP384i experiments applied a series of single bolus applications (5 μl “puff”) of pH 6.5 solution with intervening periods in pH 7.4 solution to enable agonist wash-off and ASIC1a receptor recovery from desensitization. This was repeated several times in the same cell to ensure reproducible activation which is critical for subsequent pharmacology experiments. In single hole recordings from a stable CHO hASIC1a cell line grown for several days at 37°C, we observed robust inward current responses in most cells across the 384 well plate after 3–6 h of tetracycline induction, with an average current amplitude of −5 nA (Figure 8A and Table 3). Currents activated rapidly after liquid exchange and then exhibited a mono-exponential decay to baseline, likely due to a mixture of initial ligand removal and receptor desensitization in the single “puff” application format, as subsequent full wash-off occurred several seconds after pH stimulus due to the delay in moving the 384 tip assembly between liquid reservoirs and the recording site. Nevertheless, acid-induced responses were stable, as shown by a sequence of seven pH 6.5 applications followed by washout and recovery at pH 7.4 (for 2 min), evidence of the complete recovery from ASIC1a receptor desensitization in this assay paradigm (Figure 8A). Across two 384 single hole plates tested with single bolus “puff” pH applications we observed a QC success rate of 80.2 and 85.4% based on filters of > −500 pA peak current and > 100 MΩ whole-cell seal resistance at the end of the experiment. It is important to note that actual seal quality was far higher than the minimum cut-off value, with average values of 1.29 ± 0.62 GΩ and 1.12 ± 0.06 GΩ calculated at the end of each experiment from 308 to 328 cells on each test NPC-384 chip, respectively.

**TABLE 3:** Cell parameters, pH 6.5-evoked current expression and success rates for single and multi-hole chips using different liquid addition regimes.

Chip type	Pipetting technique	Rseal (GΩ)	Cm (pF)	Imax (nA)	Success rate
1X	Puff	1.12 ± 0.06	13.5 ± 0.4	−5.3 ± 0.1	328/384 (85%)
4X	Puff	0.38 ± 0.02	49 ± 1	−8.2 ± 0.1	342/384 (89%)
1X	Stacked Puff	3.65 ± 0.23	13.2 ± 0.6	−5.4 ± 0.2	289/384 (75%)

Shown are parameters for Rseal (for last sweep of the experiment), cell capacitance (Cm) and current amplitude (Imax) of the 1st sweep to pH 6.5. Success rate is calculated for fraction of cells across each 384 well plate meeting or exceeding minimum quality criteria. Using single “puff” applications of pH 6.5 solution in single hole chips, we obtained small molecule reference pharmacology data on the SP384i that were very similar to potencies seen on the other APC platforms in this study and from the literature. Amiloride inhibited peak current with an IC_50_ of 5.90 ± 0.31 μM (Mean ± S.E.M, *n* = 193) and a Hill slope of 1.20 ± 0.05, while Benzamil exhibited a slightly higher potency of 1.37 ± 0.07 μM (Mean ± S.E.M, *n* = 224) and a Hill slope of 1.07 ± 0.02 (Table 4).

**TABLE 4:** IC_50_ values for Amiloride and Benzamil using different pipetting regimes and chip types (1X single hole and 4X multi-hole).

Chip type	Pipetting technique	Amiloride		Benzamil	
		IC_50_ (μM) and n	Hill slope	IC_50_ (μM) and n	Hill slope
1X	Puff	5.90 ± 0.31 (193)	1.20 ± 0.05	1.37 ± 0.07 (224)	1.07 ± 0.02
4X	Puff	6.01 ± 0.14 (328)	1.14 ± 0.01	1.47 ± 0.03 (357)	1.12 ± 0.02
1X	Stacked Puff	10.9 ± 0.6 (192)	1.39 ± 0.02	2.48 ± 0.18 (191)	1.26 ± 0.03
4X	Stacked Puff	8.31 ± 0.23 (113)	1.24 ± 0.02	2.20 ± 0.08 (112)	1.12 ± 0.02

Shown are mean ± S.E.M, number of wells shown in parentheses. For some conditions, results from multiple plates are given.

Very similar ASIC1a biophysics, current characteristics and pharmacology were obtained with single “puff” pH responses on multi-hole NPC-384 chips with four holes (cells) per well. Current amplitude was obviously increased due to summation of current from multiple cells (Table 3), but ASIC1a current kinetics and assay stability were unchanged using a series of single bolus application of pH 6.5 (Figure 8B). The potencies of Amiloride and Benzamil (6.01 and 1.47 μM, respectively; Figures 8C,D) were similar to those obtained for Amiloride and Benzamil using single hole chips (Table 4).

Assay performance was consistently high across three multi-hole NPC chips, with a QC pass rate of 93.8, 92.2, and 82.6% based on a minimum current amplitude of −500 pA and seal resistance > 25 MΩ at the end of each experiment. As the average seal resistance value is divided across all 4 sites in each well, this QC parameter is similar to the 100 MΩ cut-off used for single hole recordings. Actual seal resistances were significantly higher in the majority of cells and averaged 0.38 GΩ (Table 3), which as explained previously is equivalent to a mean single cell seal four times this value. As expected, the QC pass rate is improved when using multi-hole chips, mainly due to fewer sites falling below the minimum current amplitude. In addition, more consistent pharmacological data can be obtained across multiple recording sites (Figure 8E). Assay performance as measured by Z’ analysis is equally good, and both APC recording formats deliver excellent plate-based screening assay statistics above the industry standard z’ cut-off of 0.5 (Table 5).

**TABLE 5:** Comparison of SP384i ASIC1a assay performance in single vs. multi-hole chips.

Chip type	Pipetting technique	Z’ value				
		*n* = 1	*n* = 2	*n* = 3	*n* = 4	*n* = 8
1X	Puff	0.62 ± 0.08	0.73 ± 0.05	0.78 ± 0.04	0.81 ± 0.04	0.86 ± 0.03
4X	Puff	0.60 ± 0.03	0.72 ± 0.02	0.77 ± 0.02	0.80 ± 0.02	0.86 ± 0.01
1X	Stacked Puff	0.63 ± 0.05	0.73 ± 0.03	0.78 ± 0.03	0.81 ± 0.02	0.87 ± 0.02
4X	Stacked Puff	0.69 ± 0.03	0.78 ± 0.02	0.82 ± 0.02	0.84 ± 0.01	0.89 ± 0.01

For each plate there were 64 wells (four columns) of positive control (incubation in pH 6.5 solution, fully desensitized response) and negative control (0.1% DMSO) solution. Z’ values were calculated with replicate number 1, 2, 3, 4 or 8 and the mean ± S.E.M of the 4 pairs of controls for each plate are shown.

**FIGURE 9:**
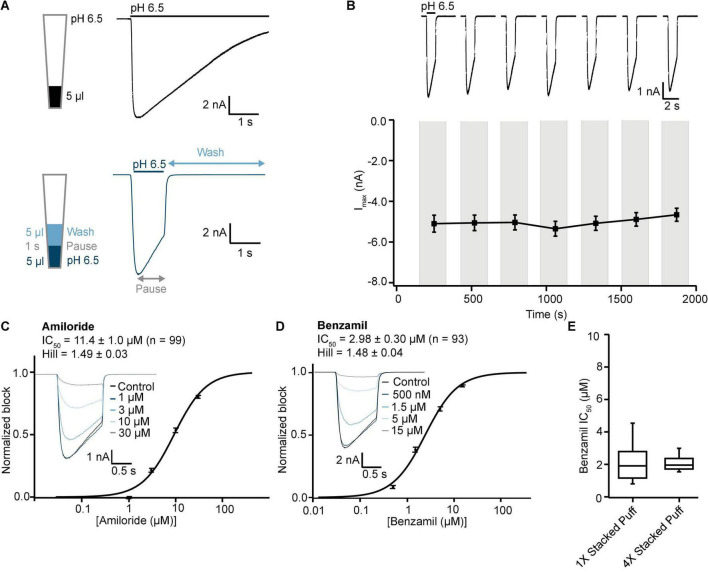
Validation of ASIC1a stacked tip assay format on SP384i. **(A)** Comparison of “puff” ligand application versus “stacked puff” format. Top left shows a schematic of the “puff” solution in the pipette tip and corresponding example of ASIC1a-mediated current with slow desensitizing phase. Bottom left shows the schematic of the “stacked puff” solutions in the pipette and an exemplar ASIC1a-mediated current with slow desensitization followed by rapid wash-off. **(B)** Stable responses in a single hole well to repeated “stacked puff” applications of pH 6.5 (top), and average current stability data from *n* = 48 wells (bottom; mean ± S.E.M). **(C,D)** Inhibition of ASIC1a responses using “stacked puff” approach by Amiloride and Benzamil with single hole chips. Raw current traces (insets) in response to pH 6.5 “stacked puff” activation after pre-incubation in indicated concentrations of drug enable construction of cumulative IC_50_ curves for Amiloride **(C)** and Benzamil **(D)**, with indicated mean, S.E.M and *n* values, and Hill slopes of 1.49 and 1.48, respectively. **(E)** There is a wider spread of IC_50_ values for Benzamil for 1X chips compared with 4X chips using the “stacked puff” application of ligand, although the mean values were not statistically different (*P* > 0.05, unpaired Student’s *t* test).

We next wanted to validate the stacked tip ligand-gated application format on the SP384i using the same CHO ASIC1a cell line and experimental conditions as above, to find the optimal protocol for studying this class of ion channel. Previous stacked tip ligand-gated receptor ion channel experiments on the Patchliner and SP384i typically use a small volume of agonist (5–10 μl) aspirated below a relatively large volume of extracellular buffer (200 or 35–45 μl, respectively) to wash-off the ligand (Milligan and Jiang, 2020; Braun et al., 2021; Obergrussberger et al., 2022). However, more recent work by us and others on the SP384 family of platforms has revealed that a counter-intuitive approach can be more effective, whereby a smaller wash-off volume (i.e., 5 μl) used to reduce bulk mixing in the well achieves faster and more complete wash-off above the recording site and reduces slow desensitization of ligand-gated ionotropic receptor responses (Figure 9A).

Using this optimized small volume “stacked” puff configuration on single hole chips revealed different current decay kinetics but identical pharmacology when compared with the “puff” addition. pH-activated currents rapidly reached a peak and then desensitized slowly, before rapidly returning to baseline upon washout and removal of the ligand (Figure 9A). Thus, it is possible to more reliably estimate peak amplitude and assess receptor desensitization rates with the stacked puff technique compared to a single puff application (e.g., Figures 8, 9A). Using the stacked puff method, ASIC1a currents were repetitively activated with pH 6.5 solution 7 times in the same cell, revealing reproducible and stable results suitable for pharmacological and drug screening (Figure 9B). The peak amplitude of ASIC1a-mediated currents evoked with the stacked puff approach was similar to that obtained with the puff approach using the same chip type (Table 3). Additionally, the reference inhibitors Amiloride and Benzamil blocked ASIC1a-mediated responses in a concentration-dependent manner (Figures 9C,D) with similar potency to that seen using single puff applications of pH on single hole and 4X multi-hole chips (Table 4), and were in excellent agreement with the literature (Leng et al., 2016). As seen with the puff addition (Figure 8E) the spread of IC_50_ values for Benzamil using 1X chips and the stacked puff application was higher than when 4X chips were used (Figure 9E).

**FIGURE 10:**
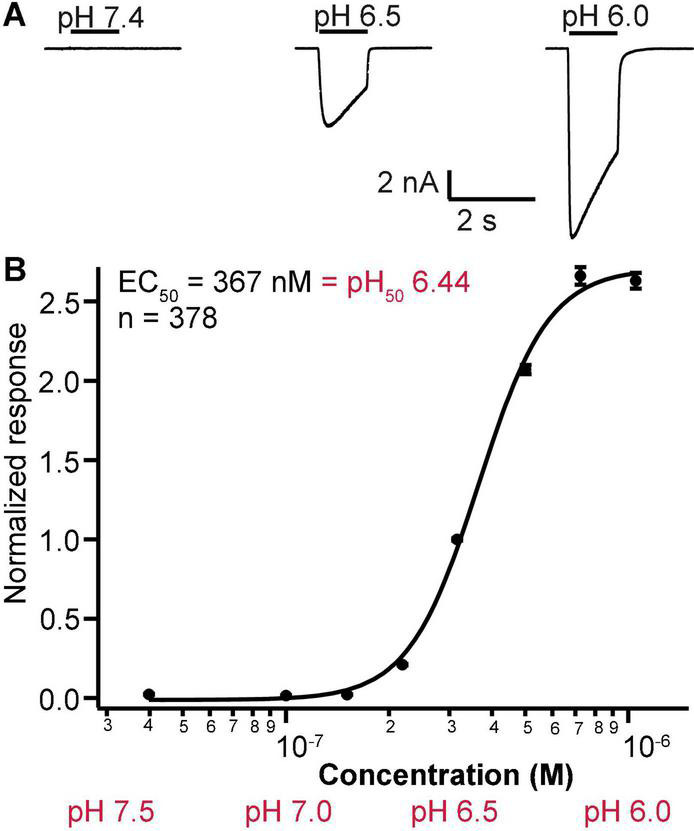
pH sensitivity of ASIC1a responses on SP384i using stacked puff format. **(A)** pH was used to activate ASIC1a responses, and decreasing pH led to increased peak amplitude (exemplar traces from different cells shown on the same scale). The pH concentration-response curve was calculated across multiple wells (each well received 1 × pH 6.5 followed by the test pH so the data was normalized to the pH 6.5 response in each well). **(B)** The pH_50_ was calculated to be 6.44. Mean ± S.E.M for each pH is shown by symbols from a maximum of 48 wells per pH concentration. 4X chips were used.

Using the optimal stacked puff format and 4X chips, application of increasingly acidic pH evoked rapidly activating and deactivating inward currents (Figure 10A), enabling a pH activation plot to be constructed (Figure 10B). To obtain the composite pH EC_50_ data, a stable current response was obtained with three applications of pH 6.5 to each well. Following this the test pH for each well was then applied, and this cycle was repeated twice with a washout at pH 7.4 between each pH stimulus to acquire a reliable pH response. For each well the test pH response was normalized to the current elicited by pH 6.5, and the pH concentration-response curve calculated from all QC-passed wells across the whole plate. In these experiments the pH which elicited half-maximal current (pH_0.5_) was 6.44 ± 0.08 (mean ± S.D., *n* = 378), in excellent agreement with literature values from manual patch clamp (Gunthorpe et al., 2001), other SP384 studies (Braun et al., 2021) and other APC platforms (data above and references herein).

## Discussion

The results from this collaborative project demonstrate the successful implementation of technically challenging ligand-gated ion channel assays on a range of low, medium and high throughput APC platforms using human ASIC1a channels as a representative target of current interest for drug discovery screening. These data offer examples of how to overcome common problems in making such recordings, such as improving current amplitude with cell culture protocols and the use of multi-hole chips and plates. We also offer a comparison of different ligand application techniques such as stacked tips that are available on different pipette and tip-based APC platforms. Importantly, the agonist (pH EC_50_) and antagonist sensitivity of ASIC1a responses are very similar across multiple APC platforms using different ligand-gated application protocols. We also utilize the current clamp capabilities available on most APC platforms to validate a traditional input resistance tracking method on the Patchliner. Finally, our data demonstrate the advantages and risks of using endogenous responses in parental cell lines as the basis for screening assays. Although we successfully replicated a manual patch clamp study of endogenous pH-gated responses in HEK cells attributed to human ASIC1a channels on APC, our extensive validation of this assay indicated an atypical pharmacological profile and low current expression which precluded its use for drug discovery screening.

### Endogenous proton-activated currents in HEK cells

It is relatively common in ion channel drug discovery to use native and immortalized cell lines, either for their endogenous human or mammalian ion channel proteins (e.g., Vetter et al., 2012) or as a system for heterologous expression of human orthologs (Rogers et al., 2016). However, the endogenous voltage- and ligand-gated ion channels in the chosen cell line are not always determined before drug discovery screening or mechanistic studies take place, raising questions over the accuracy of subsequent work. It is therefore wise to test for functional responses of the ion channel target family of interest (Gunthorpe et al., 2001; Rogers et al., 2016), profile the cell line system using molecular biology techniques (Gunthorpe et al., 2001), and if necessary knockdown the endogenous target before expressing the functional protein or mutant of interest (Vanoye et al., 2016; Borg et al., 2020; Braun et al., 2021). In the case of ASIC drug screening, the use of parental HEK cells to express mutated hASIC1a (Cullinan et al., 2019) and other human and species orthologs (Kuduk et al., 2010; Wolkenberg et al., 2011; Goehring et al., 2014) may be compromised by contamination from the innate ASIC1a channels we and others have characterized in HEK cells.

Although we were able to confirm the original manual patch clamp publication that native HEK cells express an endogenous ASIC1-like response (Gunthorpe et al., 2001), there are several features of these pH-activated currents that do not make them sufficient for a drug discovery screening assay, especially on APC platforms. Firstly, we found that pH-activated current amplitude was relatively low and that treatments to increase it led to unstable responses unsuitable for pharmacological screening. With HEK cells grown under standard 37°C conditions the mean peak inward current amplitude ranged between −334 and −567 pA for single cell manual and automated patch clamp recordings, respectively (Gunthorpe et al., 2001; Figure 1), leaving a small assay window once a minimum current threshold is applied. The lower current amplitude (and % expressors) in APC experiments may be due to loss of neuronal-like processes of adherent HEK cells that express functional ASIC1a receptor channels when cells are dissociated and suspended for use on APC platforms. In order to induce current amplitudes of sufficient size for single hole QPatch 48 experiments cells must be incubated for at least 24 h at 30°C, but this mild heat shock treatment affects ASIC1 current stability and may also trigger surface translocation of other ion channels, thereby reducing assay success rates and specificity. Thus, the trade-off between a treatment to increase current expression and maintain overall assay stability and quality failed to yield a set of experimental conditions in HEK cells that met our goal of achieving a ≥ 50% final QC success rate at the end of every single cell cumulative xC_50_ ASIC1a screening experiment. Multi-hole recordings from untransfected HEK cells proved problematic, removing an alternate approach to overcome low current amplitudes and expression rates. Although many APC users now adopt multi-hole recordings as standard, this may mask underlying issues with poorly expressing transient transfections or stable cell lines and sub-optimal cell culture, and also precludes the application of some useful QC filters such as seal resistance and Rseries monitoring. We treated single-hole and multi-hole recordings of hASIC1a currents as separate experiments to enable comparison of recording modes on the APC platforms used in this study, so that an appropriate choice of assay and chip format can be made by other users.

Secondly, we found that the pharmacological profile of the endogenous pH-gated response in HEK cells was not typical for hASIC1a receptors. Amiloride was surprisingly potent, and the moderately selective toxin Mambalgin-3 was more effective than the selective ASIC1 antagonist PcTx-1. Although we were able to replicate the low μM potency of Amiloride seen in the original manual patch clamp study (Gunthorpe et al., 2001) in our QPatch 48 recordings (2.2 vs. 2.8 μM, respectively), both values are more potent than the 10–20 μM values typically obtained in other electrophysiological studies of human and rodent ASIC1a receptors (Baron and Lingueglia, 2015; Leng et al., 2016). In contrast, the potency of 119 nM for Mambalgin-3 inhibition of endogenous pH-gated responses was close to values for human ASIC1a in previous studies (Diochot et al., 2012; Sun et al., 2018), although mambalgins are only moderately selective for ASIC1a channels, exhibiting 1.2 – 5 fold selectivity for rat ASIC1a over other ASIC1 and ASIC2 homomers, heteromers and splice variants. Also, the highly ASIC1a-selective toxin PcTx1 was only moderately effective against pH-activated currents in HEK cells, exhibiting weak, variable and inconsistent inhibition. Mean inhibition by PcTx1 at the highest tested concentration of 30 nM was < 50%, whereas the published potency against human ASIC1a is ∼3 – 13 nM (Baron et al., 2013; Cristofori-Armstrong et al., 2019). Despite the fact it can be difficult to replicate manual patch data for sticky compounds, peptides and other biological ligands on APC platforms, we followed best practice and employ protocols to minimize compound absorption or loss (e.g., glass-lined consumables throughout, BSA in final experimental solutions). As our pharmacology data faithfully replicates the known potency of Mambalgin-3 and PcTx1 against human ASIC1a responses in CHO cells on the same APC platform (Table 2), this suggests that the relative potency and efficacy of non-selective vs. selective ASIC1a ligands in HEK cells is evidence for functional expression of an atypical pH-gated receptor complex in this parental cell line.

Whilst TRP channels are present in HEK cells (Wu et al., 2000), our data rules out their contribution to endogenous pH-gated responses in HEK cells; there was no effect of the pan-TRP antagonist Ruthenium Red (Figure 2E), and Mambalgin-3 produced over 75% inhibition of whole-cell current (Figure 2B) but does not inhibit TRPV1 receptor channels (Diochot et al., 2012). Interestingly, co-assembly of ASIC1 and ENaC subunits in *Xenopus* oocytes increases the potency of Amiloride (Meltzer et al., 2007), and ENaC channels are more sensitive to inhibition by Amiloride (100 nM, Canessa et al., 1994) than ASIC1 channels, perhaps explaining the relatively high sensitivity of endogenous proton-gated responses in HEK cells to Amiloride inhibition found in this study (2.8 μM) and the 2.2 μM value reported by Gunthorpe et al. (2001). As all of the pharmacology testing was done in HEK cells grown at 30°C it is important to note that heat shock induction of intracellular chaperones may facilitate the expression of various endogenous pH-gated conductances in HEK cells, potentially leading to alterations in agonist and antagonist pharmacology. However, we observed no change in the pH EC_50_ values in HEK cells (Figure 1D) or CHO cells (data not shown) after 30°C treatment, suggesting that low temperature treatment of HEK cells predominantly upregulated ASIC1-containing receptor channel complexes, or alternatively that ASIC and other degenerin family ion channels such as ENaC may have been increased but all share a similar pH sensitivity (Meltzer et al., 2007).

### Application of conductance-tracking in a ligand-gated ion channel automated patch clamp assay

Prior to the development and widespread utilization of whole-cell patch clamp techniques in ion channel research, neurotransmitter-gated receptor responses were often studied using sharp microelectrodes and current clamp monitoring of cell input resistance to track changes in conductance in response to agonist application by iontophoresis or bath application (e.g., Hiruma and Bourque, 1995). This technique has now been supplanted but a recent study from an industry group detailed a low throughput electro-optical version of the input resistance tracking method using field electrodes to apply current pulses and fast voltage-sensing dyes to measure membrane potential changes in populations of heterologous cell lines (Menegon et al., 2017). In theory this optical approach could be scaled up to 96 and 384 well plate-based imaging systems used in ion channel drug discovery, but we wanted to demonstrate that the current clamp features available on most APC platforms could be used to design a higher throughput conductance-tracking assay to study ligand-gated ionotropic receptor targets relevant to ion channel drug discovery.

Using the Patchliner as our proof-of-concept platform, we successfully developed and validated an APC input resistance tracking assay for the fast ligand-gated ASIC1a receptor channel (Figure 4). After careful optimization of current clamp protocols and choice of a holding potential that allowed for reliable detection of changes in input resistance in response to a series of current injections, it was possible to accurately track changes in membrane conductance after the application of pH agonist. Our current clamp data reveal pH EC_50_ and Benzamil IC_50_ values in close agreement with those obtained using fast ligand application under voltage clamp on the Patchliner and other APC platforms. This is the first demonstration to our knowledge for implementation of the traditional current clamp conductance tracking technique to study a ligand-gated receptor channel on a multi-well APC platform, demonstrating the untapped utility of these devices.

### ASIC1a ligand-gated screening assay validation on automated patch clamp platforms

While voltage-gated ion channel screening is well served by the gigaseal recording qualities and sophisticated biophysical features of current APC platforms, successful implementation of robust pharmacological assays for ligand-gated receptor channels has been more challenging. Early protocols on low and medium throughput platforms and initial models of 384 devices utilized slow and sequential additions of agonist and wash-off solutions due to the need for the tip or pipette to return to a reservoir of external solution and then back to the recording site to wash-off the agonist, which can lead to profound receptor desensitization. Such assays run the risk of screening against a non-physiological state of the ligand-gated ionotropic receptor of interest, and can also flatter users by revealing an agonist EC_50_ for the desensitized state which is more potent than the activated state. Numerous ligand-gated receptor channels of therapeutic interest exhibit such desensitization, including ASIC channels, α7 and other nAChRs, as well as GABA_*A*_ receptors, NMDA, kainate and AMPA GluRs, and P2_*X*_ receptors. APC vendors have now developed new ligand-gated assay features with faster application speeds and the ability to stack different solutions within each tip, so we sought to compare the same ligand-gated hASIC1a receptor channel assay across multiple APC platforms and test the efficacy of various optimization strategies.

A key parameter for the accuracy and reliability of a ligand-gated ionotropic receptor assay is the agonist EC_50_, as this value can be compromised by compound absorption, slow or partial access to the ligand-binding pocket, and receptor desensitization. In the case of ASIC1a the pH dependence is steep (Hill slope > 5) so even small deficits in the assay format and liquid application and wash-off protocols can significantly affect this value. It was therefore reassuring to see very similar pH EC_50_ values obtained using the same CHO ASIC1a cell line on the Patchliner with stacked pipette tips (6.68) and on the QPatch 48 using a bolus pipette application (6.62). These values are comparable to pH EC_50_ potencies published for hASIC1a on the QPatch and SP384 family of APC platforms (Braun et al., 2021; B’Sys, 2022). A slightly more acidic pH EC_50_ of 6.44 was obtained on the SP384i with a stacked puff application from plastic tips, but it should be noted that multi-hole chips and a different CHO cell line were used in these experiments. It was clear that ASIC1a receptor desensitization occurred over a second or so after current activation and prior to ligand wash-off with the bolus applications on the SP384i, and that this could be minimized using the various stacked tip protocols now available on most APC platforms. All of these APC pH EC_50_ values are significantly different from the much more acidic EC_50_ value of 5.6 obtained for hASIC1a in *Xenopus* oocytes where fast perfusion and complete solution washout is problematic (Borg et al., 2020).

Although there is significant desensitization of hASIC1a responses during more prolonged and acidic pH applications, seen in both bolus or “puff” applications (e.g., Figures 1A, 3A, 6A, 9), ASIC1a receptor ion channels recover quickly from desensitization upon return to neutral pH. Thus, we saw no obvious differences in pH EC_50_ values across the Patchliner, QPatch 48 and SP384 APC platforms as care was taken to effectively remove acidic solution during wash-off and allow the receptors to recover from desensitization with repeated periods in pH 7.4. The same rapid recovery from desensitization may not be true for other types of ligand-gated ion channels (e.g., α7 nAChR, neuronal P2_*X*_ receptors, GluRs), in which case it becomes useful and perhaps essential to employ APC assays with stacked ligand and wash solutions to achieve effective ligand-gated assays with reduced desensitization, current rundown and altered pharmacology.

Once we had established the correct pH EC_50_ value for each APC platform and ASIC1a cell line it was possible to pharmacologically validate the assay using a toolbox of reference compounds, including both small molecules and peptide toxins that are common ligand modalities in ion channel drug discovery. Benzamil was tested across all APC platforms and exhibited very consistent potencies when tested using a bolus pipette application (QPatch 48) or stacked tip application (Patchliner, SP384i). Focusing on data from single hole recordings, we obtained an IC_50_ for Benzamil of 3.65 μM on Patchliner (Figure 3C), 4.66 μM on QPatch 48 (Figure 6C and Table 2), and 1.37 and 2.48 μM on SP384i using puff or stacked puff applications (Table 4), respectively. Amiloride was used as a positive control for the medium throughput QPatch 48 and high throughput SP384i platforms, exhibiting an IC_50_ of 7.62 μM on the former (Figure 6C and Table 2) and values of 5.90 and 10.9 μM on the latter with puff or stacked puff applications (Table 4), respectively. The potencies of Amiloride and Benzamil across these APC platforms (Tables 2, 4) align well with published values of 3.5 and 13.5 μM, respectively (Leng et al., 2016).

A comprehensive ASIC compound toolbox was tested on the QPatch 48 as we wanted to validate the CHO ASIC1a cell line on this platform prior to a fragment screen (Rogers et al., 2021b). We found excellent agreement between the nM potencies of the selective ASIC1a toxins PcTx1 and Mambalgin-3 on the QPatch 48 compared to literature values (Table 2), confirming the ability to reliably detect the activity of biological ligands in APC assays if suitable steps are taken to minimize peptide absorption. PcTx1 binds within the “acidic pocket” in the extracellular domain of ASIC channels, and as such is a state-dependent modulator as its affinity can be strongly affected by local pH (Baron et al., 2013). Pre-incubating CHO cells in the presence of PcTx1 at pH 7.3 was sufficient to allow efficient binding, and the potency of 3 nM we obtained on the QPatch 48 (Figure 6D and Table 2) was nearly identical to published values of 0.9 – 3 nM for human ASIC1a channels (Escoubas et al., 2000; Cristofori-Armstrong et al., 2019; Braun et al., 2021). Whilst there is a paucity of data for Mambalgin-3 against human ASIC1a channels as most published data utilized mammalian orthologs (Diochot et al., 2012; Sun et al., 2018), this toxin only differs from Mambalgin-1 at one non-critical amino acid residue and it is assumed to exhibit similar potency. Indeed, the very similar potencies of Mambalgin-3 on QPatch 48 of 119 and 160 nM against endogenous pH-gated responses in HEK cells (Figure 2B) and human ASIC1a stably expressed in a CHO cell line (Table 2) align very closely with quoted values of 127 or 197 nM for Mambalgin-1 tested against human ASIC1a channels (Diochot et al., 2012; Sun et al., 2018). Unlike PcTx1, Mambalgins are no longer thought to bind within the “acidic pocket” of the ASIC1a receptor but nearby on the “thumb,” and thereby disrupt (reduce) proton sensitivity of activation (Baron et al., 2013; Cristofori-Armstrong and Rash, 2017). In this way Mambalgins may bind to both the closed and desensitized/inactivated states of ASIC1 receptor channels but don’t alter their desensitization pH sensitivity.

Several small molecule antagonists of ASIC receptor channels were also tested on the QPatch 48 in preparation for a drug discovery screen. The clinical drugs Amantadine and Memantine are low molecular weight hydrophobic amines that inhibit various ASIC receptor channels with low potency (Nagaeva et al., 2016; Shteinikov et al., 2018), and we found IC_50_ values of 510 and 315 μM against human ASIC1a, respectively (Table 2). Although A-317567 is not selective for ASIC1a as it was originally designed by Abbott as an ASIC3 modulator (Dubé et al., 2005) and used as a scaffold to develop further analogues against this target by Merck and Co., Inc., (Kuduk et al., 2010), A-317567 is a relatively potent drug discovery compound that can be used to benchmark screening assays designed to discover new selective and potent inhibitors of ASIC1a receptor channels. Compared to published data that A-317567 inhibits “ASIC1-like” pH-activated currents in rat dorsal root ganglion neurons with a potency of 2 μM (Dubé et al., 2005), we found that human ASIC1a channels exhibited an IC_50_ of 660 nM (Figure 6C and Table 2), with the small difference in activity likely attributable to differences in activating pH and cell type. Significantly, a close analogue of A-317567 developed by Merck and Co., Inc., was found to inhibit ASIC1a currents with an IC_50_ of 450 nM (Kuduk et al., 2010). In summary, our pharmacological validation data highlights the current lack of potent and selective small molecule inhibitors of hASIC1a, and demonstrate the need for further virtual and compound library screens to discover novel and effective ASIC1a receptor channel modulators to treat peripheral and CNS diseases.

All APC platforms offer the choice between single hole well/single cell recording chips, and multi-hole or population patch clamp recordings. Single hole chips and plates are preferred for rigorous biophysical recordings of voltage-gated ion channels as they allow for application of compensation circuits and tracking of single cell properties for QC purposes, and enable an accurate assessment of variation in cell line characteristics during assay optimization. Multi-hole recordings are favored as they increase assay success rate and reduce screening costs, and can compensate for sub-par cells or difficult-to-express ion channel targets. We were interested to assess what difference, if any, that single vs. multi-hole APC recordings would make for a ligand-gated receptor channel such as ASIC1a due to potential differences in liquid application and wash-off for a single cell compared to an array of cells across the bottom of a multi-hole recording well. Significantly, we did not find any obvious differences in the pharmacological potency of reference small molecule compounds and peptide toxins tested in single vs. multi-hole format on the QPatch 48 (Figure 7) and SP384i (Table 4). Also, multi-hole recordings can deliver ligand-gated receptor ion channel assays with a higher success rate (Figure 7) and thus improved screening efficiency and lower costs, as well as more consistent pharmacological results (Figures 8E, 9E).

In summary, we demonstrated that it is possible to implement robust and reliable medium and high throughput APC screening assays for a challenging ligand-gated receptor ion channel. This was achieved on three different APC instruments, Patchliner, QPatch 48 and SP384i, by careful design of an ASIC1a assay format that maximized current stability and minimized desensitization during repeated pH activations (Figures 5B, 8A,B, 9B), enabling accurate pharmacological sensitivity. The need for a more efficient and cost-effective ASIC1a drug screening assay is topical (Bordon et al., 2020), as previous efforts to bring ASIC modulators to the clinic for various therapeutic indications have failed for various reasons (Wolkenberg et al., 2011; Heusser and Pless, 2021). Previous screening efforts used manual patch techniques and low throughput 16 channel APC devices, and traditional 384 plate-based fluorescence imaging assays to detect cation flux through ASIC1a receptor channels has proven challenging due to the rapid activation and profound desensitization of these channels that is hard to overcome on systems with slow or limited liquid addition and washout capabilities. Thus, our demonstration of improved, robust and efficient ligand-gated ionotropic receptor screening assays on a range of APC platforms may foster renewed efforts to discover potent, selective and efficacious modulators of human ASIC1a channels.

## Data availability statement

The original contributions presented in this study are included in the article, further inquiries can be directed to the corresponding author.

## Author contributions

SM carried out the Patchliner experiments designed by MR, and undertook the data analysis and figure creation. RT, JR, and RK designed and carried out the QPatch 48 experiments and data analysis and helped prepare the figures. TG and NB carried out the SP384i experiments and data analysis, supported by IR-W. AO and NB prepared the figures. AO and MR collected, organized, and interpreted the results, and co-wrote the manuscript. MR conceptualized the study and coordinated the project and dissemination of the results. All authors contributed to the article and approved the submitted version.
